# New-Onset Arthritis Following COVID-19 Vaccination: A Systematic Review of Case Reports

**DOI:** 10.3390/vaccines11030665

**Published:** 2023-03-15

**Authors:** Jie Liu, Hui Wu, Sheng-Li Xia

**Affiliations:** 1 Shanghai Jiao Tong University School of Medicine Library, No. 280, South Chongqing Road, Shanghai 200025, China; 2 Department of Orthopedics, Shanghai University of Medicine & Health Sciences Affiliated Zhoupu Hospital, 1500 Zhoupu Zhouyuan Road, Pudong New Area, Shanghai 201318, China

**Keywords:** coronavirus disease 2019 (COVID-19), arthritis, adult-onset Still’s disease (AOSD), rheumatoid arthritis (RA), reactive arthritis (ReA), bursitis, synovitis

## Abstract

Coronavirus disease 2019 (COVID-19) vaccine has effectively suppressed the spread of the severe acute respiratory syndrome coronavirus 2 (SARS-CoV-2) and alleviated its symptoms, but there are also many adverse events. Joint diseases caused by COVID-19 vaccine have been reported in many studies. Some are well-controlled arthritis patients who developed arthritis after COVID-19 vaccination, while others are new-onset joint pain and swelling problems after COVID-19 vaccination. The purpose of this systematic review is to examine the literature reports in existing databases and analyze the incidence of new-onset arthritis after COVID-19 vaccination. We included 31 eligible articles and described 45 patients, ranging in age from 17 to over 90, with more females than males. The majority (84.4%) of patients received the adenovirus vector vaccine (ChAdOx1) and the mRNA-based vaccine (BNT126b2 and mRNA-1273). Most (64.4%) patients developed joint-related symptoms after the first dose of vaccine, and 66.7% developed symptoms within the first week of vaccination. The joint symptoms involved were mainly joint swelling, joint pain, limited range of motion, and so on. A total of 71.1% of the patients involved multiple joints, both large and small; 28.9% of patients involved only a single joint. Some (33.3%) patients were confirmed by imaging, and the most common diagnoses were bursitis and synovitis. Two nonspecific inflammatory markers, erythrocyte sedimentation rate (ESR) and C-reactive protein (CRP), were monitored in almost all cases, and all patients showed varying degrees of increase in these two markers. Most of the patients received the treatment of glucocorticoid drugs or nonsteroidal anti-inflammatory drugs (NSAIDs). Clinical symptoms markedly improved in most patients, with 26.7% making a full recovery and no relapse after a few months of follow-up. To determine whether there is a causal relationship between COVID-19 vaccination and the triggering of arthritis, large-scale and well-controlled research studies are needed in the future to verify this relationship and to further study its pathogenesis in detail. Clinicians should raise awareness of this complication with a view to early diagnosis and appropriate treatment.

## 1. Introduction

In December 2019, a novel coronavirus strain, called severe acute respiratory syndrome coronavirus 2 (SARS-CoV-2), emerged globally [1]. Over the next few months, SARS-CoV-2 spread to many countries around the world, affecting hundreds of millions of people. In response to the spread of the pandemic, several companies around the world have developed vaccines against coronavirus disease 2019 (COVID-19), such as BNT162b2 (by BioNTech-Pfizer), mRNA-1273 (by Moderna), ChAdOx1-nCoV19 (by AstraZeneca-Oxford), CoronaVac (by Sinovac), BBV152/Covaxin (by Bharat Biotech), and Sputnik-V (by VABIOTECH). Currently, the vaccine has been widely available around the world, and most people have received three or four doses of the vaccine, which has effectively suppressed the spread of SARS-CoV-2 and reduced its symptoms.

However, there are also many common adverse events to COVID-19 vaccines, including pain at the injection site, fever, headache, nausea, and vomiting, all of which may develop after the first and/or second administration [2]. Observed adverse events also include myocarditis [3], shoulder injury [4], reactivation of varicella zoster virus (VZV) [5], and thromboembolism [6]. Cases of vaccine-induced arthritis have also been reported in the last two years, in patients with previously well-controlled rheumatism or adult-onset Still’s disease (AOSD), which relapsed after COVID-19 vaccination [7,8], or in patients who had no medical history of joint disease or rheumatism but had new-onset joint pain and swelling after vaccination [9].

To determine the most common characteristics of new-onset arthritis occurring after COVID-19 vaccination and its clinical characteristics, we conducted a systematic review of new-onset arthritis cases after COVID-19 vaccination reported in the literature, with a view to providing a reference for clinicians.

## 2. Methods

### 2.1. Search Strategy

We followed PRISMA guidelines for this study. Two researchers systematically searched PubMed, Embase, Web of Science, and Cochrane Library databases for literature on arthritis after COVID-19 vaccination. The search term was as follows: “(COVID-19 vaccin*) OR (SARS-COV2 vaccin*) OR (SARS-COV-2 vaccin*) AND (Arthritis OR arthrophlogosis OR Felty Syndrome OR Still’s Disease OR Arthropathy OR Spondylarthritis)”. All relevant studies up to 1 January 2023 were searched. There were no restrictions on study design, geographic region, or language. Discrepancies in the literature retrieval process were resolved by a third researcher.

### 2.2. Selection Criteria and Exclusion Criteria

The studies we included provided data on cases of arthritis following at least one dose of COVID-19 vaccine. All study designs were considered eligible for inclusion. Studies of patients who had arthritis before vaccination with recurrent or aggravated arthritis after vaccination were excluded. Review articles, abstracts submitted at conferences, and non-peer-reviewed articles did not meet the criteria for inclusion. In vitro and animal studies were excluded.

### 2.3. Data Extraction and Handling

The data were screened and reviewed independently by two researchers, and any disagreements were discussed and resolved by a third researcher. The following information of the patients was collected: (1) basic information: the first author’s surname, country, and publication date; (2) patients’ characteristics: age and gender; (3) information on COVID-19 vaccination: types and doses of vaccines; (4) incidence of arthritis after vaccination: onset days after vaccination, clinical manifestations and physical examination, imaging diagnosis, laboratory tests, name of diagnosis, treatment, and outcome. 

### 2.4. Quality Assessment

The quality of the case reports was assessed by using the modified Newcastle-Ottawa Scale (NOS) and classified as unsatisfactory (0–3 points), satisfactory (4–5 points), good (6–7 points), and very good (8–9 points) [10]. The NOS scale consists of three dimensions and eight items: four items for object selection, one item for intergroup comparability, and three items for outcome measurement. The assessment was conducted independently by two researchers, and discrepancies were resolved by consensus.

### 2.5. Data Synthesis and Analysis

Descriptive statistics were used to summarize the data, and percentage was used to analyze the proportion of data. Quantitative variables were presented as mean ± standard deviation, and qualitative variables were described as ratios or percentages. 

## 3. Results

### 3.1. Screening Procedure

**Figure 1:**
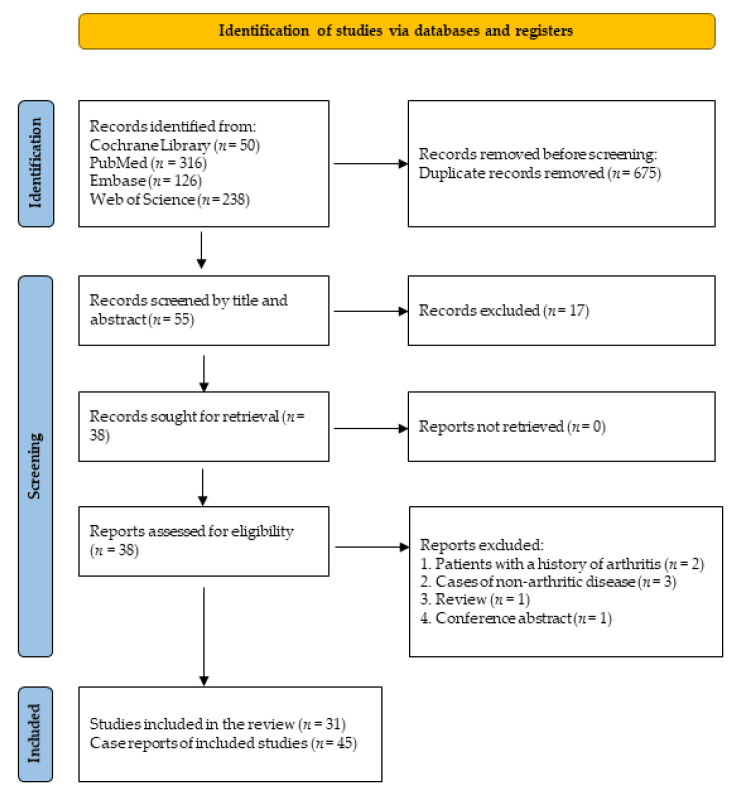
PRISMA flow diagram.

By using the aforementioned retrieval strategy, 316 publications that may meet the requirements were retrieved from PubMed, 238 from Web of Science, and 126 from Embase. Fifty trials and two reviews (both were irrelevant) were retrieved from the Cochrane Library. After de-duplication and manual screening, 55 relatively relevant articles were obtained. After further reading of the full text, reviews and literature on non-new-onset arthritis were excluded, and 31 studies that met the screening criteria were identified (Figure 1).

### 3.2. Study Results

Patients’ data information was extracted through literature screening and review, as shown in Table 1.

There were 45 new-onset cases of arthritis reported out of 31 studies, from the United States, the United Kingdom, France, Australia, the Czech Republic, Brazil, Thailand, Japan, China, etc., in which 29 cases were in Asia, 6 cases in Europe, 9 cases in the Americas, and 1 case in Oceania.

**Figure 2:**
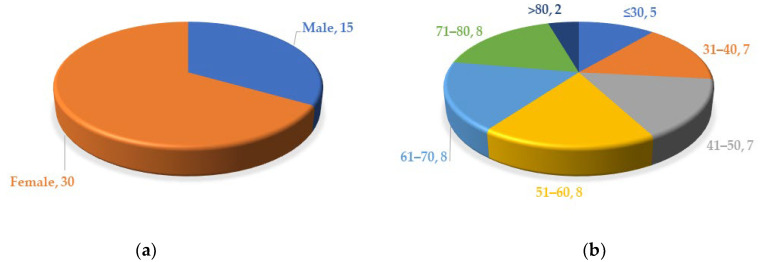
(**a**) Gender distribution of patients with arthritis; (**b**) age distribution of patients with arthritis.

We found a total of 45 cases of COVID-19-vaccine-induced arthritis (no previous medical history of arthritis) reported. There were 15 males and 30 females (Figure 2a). Patients ranged in age from 17 to over 90, including 5 patients less than or equal to 30 years old (the youngest was 17 years old), 7 patients aged 31–40 years, 7 patients aged 41–50 years, 8 patients aged 51–60 years, 8 patients aged 61–70 years, 8 patients aged 71–80 years, and 2 patients older than 80 (Figure 2b).

**Figure 3:**
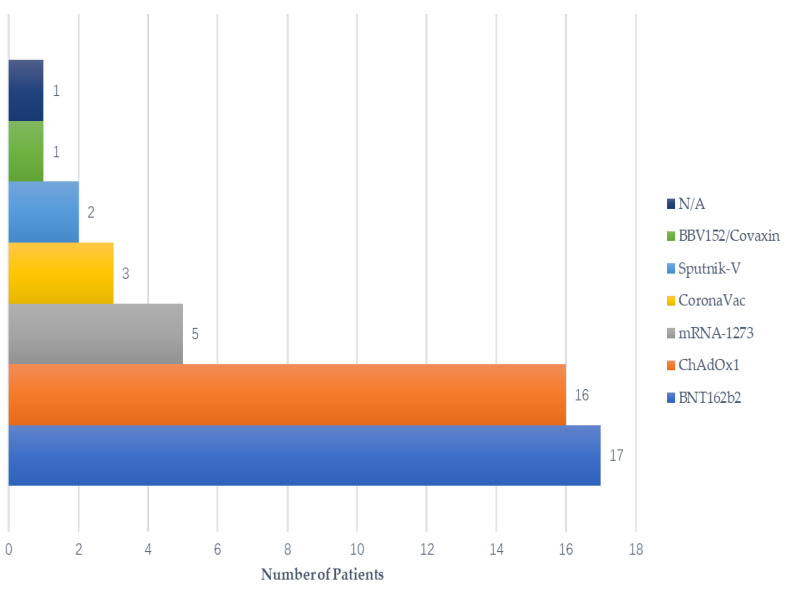
Vaccine type by onset of symptoms.

The majority of patients received the ChAdOx1 vaccine (16/45, 35.6%) and BNT162b2 vaccine (17/45, 37.8%). Five patients (11.1%) received mRNA-1273, three patients (6.7%) received the CoronaVac vaccine, and two patients (4.4%) received the Sputnik-V vaccine. One case was inoculated with BBV152/Covaxin and one with an unknown vaccine (not mentioned in the case report) (Figure 3).

Most patients (29/45, 64.4%) developed symptoms after the first dose of vaccine, while some (11/45, 24.4%) developed symptoms after the second dose; one patient developed arthralgia symptoms only after the third dose of the vaccine.

**Figure 4:**
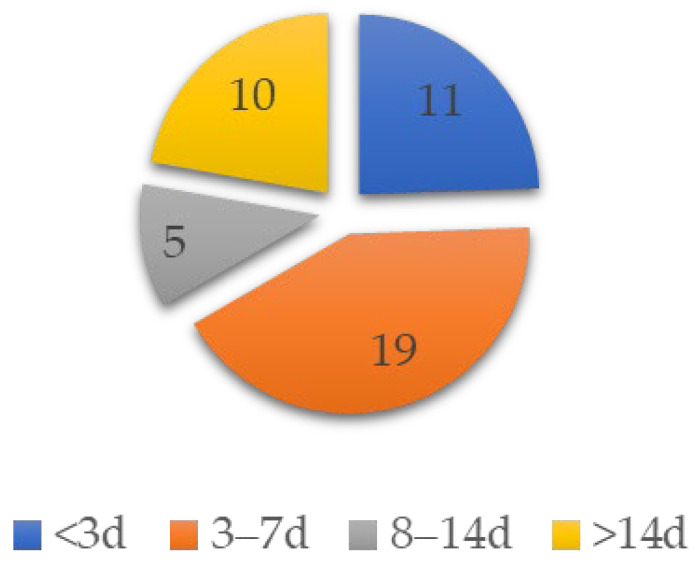
Patient numbers by onset days of symptoms.

Most patients (30/45, 66.7%) developed joint-related symptoms within 1 week of vaccination, including 11 (24.4%) within 3 days after vaccination and 19 (42.2%) within 3–7 days after vaccination. A total of 5 patients (11.4%) developed symptoms 8–14 days after vaccination; however, 10 patients (22.2%) developed symptoms 2 weeks after vaccination, with the longest case of arthralgia occurring 8 weeks after vaccination (Figure 4).

The main clinical symptoms were joint swelling, joint pain, and limited joint range of motion, accompanied by other symptoms including fever, headache, fatigue, rash, chest pain, myalgia, abdominal pain, lymph node enlargement, and spleen enlargement.

The joints involved can be single or multi-joint, bilateral or unilateral, large or small. Most cases (32/45, 71.1%) involved multiple joints, while the remaining 13 cases (28.9%) involved only a single joint. Upon further review of specific joint involvement, 12 cases were observed in the whole body, 9 in the knee, 13 in the shoulder and elbow, 2 in the chest, 4 in the sacroiliac joint, 6 in the ankle joint, and 10 in the hand joint. Bilateral joints were involved in 30 patients (30/45, 66.7%), and unilateral joints were involved in 15 patients (15/45, 33.3%). The average age of patients with bilateral joint symptoms was 54 years old, with twice as many women as men. Symptoms appeared mostly after the first dose of vaccine, in joints both large and small. The average age of patients with unilateral joint symptoms was 56 years old, and most of them were female. Symptoms appeared mostly after the first dose of vaccine, often in large joints (Table 2).

There were 15 (33.3%) radiographically confirmed cases of arthritis, and the most common diagnoses were bursitis and synovitis.

Two nonspecific inflammatory markers, erythrocyte sedimentation rate (ESR) and C-reactive protein (CRP), were monitored in almost all cases, and all patients showed varying degrees of increase in these two markers. Some patients were also examined for white blood cell (WBC), anti-immunoglobulin E (IgE), immunoglobulin G (IgG), rheumatoid factor (RF), and other related indicators, and a few patients also underwent joint puncture. Some patients were also tested for autoimmune markers, such as antinuclear antibodies (ANA), anti-citrullinated peptide antibodies (ACPA), cyclic citrullinated peptide (CCP), and so on, but just a few of them had abnormal figures, including six RF-positive patients, five ANA-positive patients, three ACPA-positive patients, one HLA-B27-positive patient, and one patient with significantly elevated interferon-beta (IFN-β) (Table 3).

Twelve patients were diagnosed with AOSD, eight with rheumatoid arthritis (RA), five with reactive arthritis (ReA), three with septic arthritis (SA), three with inflammatory arthritis (IA), one with erosive arthritis, one with peripheral spondyloarthritis (SpA), and five with arthritis of unspecified type.

All patients received treatment. For joint symptoms, most patients received glucocorticoid drugs (betamethasone, methylprednisolone, prednisolone (PDN), and dexamethasone), used in 26 patients, or nonsteroidal anti-inflammatory drugs (NSAIDs) (naproxen and celecoxib), used in 13 patients. Methotrexate was used in 3 patients, and antibiotics in 2 patients with SA. Monoclonal antibodies or physiotherapy were used in individual patients. The duration of treatment ranged from 2 days to 206 days.

Clinical symptoms markedly improved in most patients, and 12 patients (26.7%) made a full recovery with no relapse after a few months of follow-up. Three patients did not report treatment outcomes. No adverse outcomes were reported in any of the cases reviewed.

### 3.3. Quality of the Studies

Studies were assessed by using the modified NOS. Scores ranged from 6 to 9, with a median of 7.8. Three studies were given full ratings of 9 scores, twenty-one studies received 8 scores, one study was awarded 7 scores, and six studies had 6 scores. Table 4 shows the NOS quality scores of the included studies.

## 4. Discussion

In this systematic review, we studied newly induced arthritis following COVID-19 vaccination. Our results showed that the age of the patients ranged from 17 to 90 years old, with more women than men. Most patients received the adenovirus vector vaccine (ChAdOx1) and the mRNA-based vaccine (BNT126b2 and mRNA-1273). The joint symptoms were manifested mainly as joint swelling, joint pain, and limited range of motion. Bilateral joints and unilateral joints were both involved. The most common diagnoses confirmed by imaging were bursitis and synovitis. Two nonspecific inflammatory markers, ESR and CRP, were increased to varying degrees. In treatment with glucocorticoid drugs or NSAIDs, the clinical symptoms were obviously improved.

Arthritis refers to an inflammatory disease caused by inflammation, infection, degeneration, trauma, or other factors that occurs in the joints and surrounding tissues of the human body. The etiology of arthritis is complex, related mainly to autoimmune reaction, infection, metabolic disorders, trauma, degenerative diseases, and other factors. Common clinical arthritis includes osteoarthritis, RA, ReA, ankylosing spondylitis, gouty arthritis, and so on.

All 45 patients in this systematic review developed symptoms such as joint pain and swelling after vaccination, and most of them were accompanied by symptoms such as fever. Twelve of the patients developed AOSD-like syndromes, including fever, transient salmon-pink maculopapular rashes, arthritis, and neutrophil-dominated leukocytosis. The etiology of AOSD is currently unknown, but the immunopathogenesis of AOSD is believed to be caused by genetic predisposition and environmental triggers. Abnormal activation of the innate and adaptive immune system leading to the uncontrolled production of cytokines, including interleukin (IL)-1β, IL-6, IL-18, and tumor necrosis factor (TNF)-α, has been considered to be the basis of the pathogenesis of AOSD [41]. SARS-CoV-2 infection could also induce cytokine storms driven by IL-6, IL-1α, IL-1β, and TNF-α [42], and the pathogenesis of both is similar. Multiple case reports have shown that vaccination against COVID-19 can induce a strong immune response [43,44]. However, it is not possible to distinguish whether an adverse effect of vaccination is the induction of new-onset AOSD or whether it is actually a relapse of existing AOSD that plays a major part in this mechanism. Chua [37] believes that more studies and case reviews are needed to investigate the detailed mechanisms and to examine the suitability by using the same diagnostic criteria in different settings. Patients diagnosed with AOSD were treated with glucocorticoid and/or tocilizumab and/or methotrexate in the cases reviewed in this systematic review. Symptoms improved significantly after treatment. After one to two months’ follow-up, most patients showed good recovery and laboratory parameters returned to normal. No recurrence was observed in patients after six months’ follow-up. The prognosis of AOSD is good, but it is easy to relapse. It is recommended that patients gradually stop taking drugs after recovery and follow up for about half a year.

Eight patients were diagnosed with RA. Several patients [9,31] were clinically similar to polymyalgia rheumatica (PMR), RA, or other systemic inflammatory arthritis. These patients were in good health before receiving the COVID-19 vaccine and reported no symptoms of rheumatic autoimmune disease. However, it is not known whether the patients were previously asymptomatic RA-positive, because RF or ACPA were not tested before vaccination as they had no previous joint complaints [26,34]. Another potential possibility is that asymptomatic SARS-CoV-2 infection can cause rheumatic manifestations. In these cases, some patients underwent a serological anti-SARS-CoV-2 rapid test (COVID-19 IgG/IgM antibody test) and were negative [13], while others did not undergo the SARS-CoV-2 antibody test. Long-term longitudinal studies may therefore shed more light on this hypothesis and enhance monitoring of global vaccination programs to reveal the true extent of autoimmune manifestations after vaccination. As for the pathogenesis, Watanabe [26] believes that mRNA from COVID-19 vaccine is an effective inducer of a proinflammatory cytokine response, and these cytokines may mediate the autoimmune response after COVID-19 vaccination. The serum concentrations of type I IFN, IL-6, and TNF-α were significantly reduced when patients were induced to remission by methotrexate and tocilizumab, suggesting that the production of type I IFN, IL-6, and TNF-α induced by the novel coronavirus vaccine may be associated with cases of newly onset RA. In the patients with RA and PMR investigated in this systematic review, methotrexate and/or glucocorticoid and/or tocilizumab were used to induce remission, and symptoms gradually disappeared. Some patients were followed up for 10 months to complete remission, but some patients had recurrent attacks after drug reduction, so long-term follow-up is recommended.

Five patients were diagnosed by doctors as ReA, presenting with clinical symptoms in the knee [11,27], shoulder/elbow [12], and facet joints of the hand (e.g., wrist and metacarpophalangeal and proximal interphalangeal joints) [25]. All symptoms appeared within five days after vaccination. Treatment in all cases reported to date has included corticosteroid application. After follow-up, all patients recovered completely without any recurrence or consequences. ReA was rare, and the mechanism was not fully understood, but it may be caused by inactivated viruses or adjuvants found in vaccines.

Three other patients were diagnosed with SA [15,17,32], which is a less common and more serious complication, probably due to improper vaccination practices, according to the analysis of the authors of the case reports. Invasive treatment of joint irrigation and debridement is required. Therefore, vaccine managers should be careful to use aseptic techniques when vaccinating patients to reduce the risk of septic arthritis.

Although the percentage of new-onset arthritis was not calculated as patients with a history of arthritis were not included in the systematic review, according to an international study, Lisa [45] conducted an online survey of 5619 adults with systemic rheumatic disease (SRD) for adverse events following COVID-19 vaccination, among which there were RA (1701, 30.3%), psoriatic arthritis (PsA) (304, 5.4%), and ankylosing spondylitis (AS) (291, 5.2%). Disease flares requiring changes in treatment following COVID-19 vaccination were reported by 4.9% of the population respondents. The prevalence of flare was higher among those with PsA (7.9%) and PMR (8.1%). It has been demonstrated that there are not many arthritis disease flares requiring changes in treatment. Therefore, new-onset post-vaccination arthritis is a rare occurrence compared to global vaccination. The risks are far outweighed by the benefits of vaccines in preventing COVID-19 morbidity and mortality, but the presence of this complication needs to be brought to the attention of the clinician.

The systematic review provides a comprehensive overview of the currently available literature and a rigorous quality assessment of the included studies. However, there are still limitations to our study. We have reviewed only the case reports currently available, which hinders the validity and scope of the conclusions that can be drawn. These studies are particularly vulnerable to the risk of overinterpretation and selection bias. Therefore, causality cannot be inferred.

## 5. Conclusions

Considering that the patients with new arthritis in this systematic review have been vaccinated with COVID-19 vaccine for a period of time (mostly within a week), and how their mechanisms correlate with the pathogenesis of these diseases, it is speculated that there may be a certain relationship between COVID-19 vaccination and the triggering of arthritis. Clinicians should raise awareness of this complication with a view to early diagnosis and appropriate treatment, and further monitoring of the long-term prognosis of the disease is needed. However, even if there is a cause-and-effect relationship, related complications are relatively rare. Joint symptoms have improved or have been completely cured after treatment, which may be a transient response to the vaccine. In the future, large-scale and well-controlled research studies are needed to verify this relationship and further study its pathogenesis in detail.

## Figures and Tables

**Table 1:** Characteristics of new-onset arthritis after COVID vaccine.

Author (Year)	Country	Gender	Age	Vaccine Type	Dosage	Onset	Symptoms of Joint	Imaging Diagnosis	Laboratory Tests	Final Diagnosis	Treatment	Outcome
An, 2021 [11]	China	F	23	Sinovac (CoronaVac)	1st	3 d	Swollen and painful left knee joint	Moderate knee effusion	ESR 32 mm/h CRP 15.0 mg/L	ReA	Compound betamethasone	Swelling and pain nearly disappearing
Baimukhamedov, 2021 [12]	Kazakhstan	M	58	VABIOTECH (Sputnik-V)	2nd	5 d	Mild discomfort in left elbow joint, pain upon movement, and joint swelling	Moderate effusion in the left elbow fossa and a small shoulder–elbow joint synovitis	ESR 18 mm/h CRP 2.2 mg/L	Post-vaccination arthritis	NSAIDs and physiotherapy Diprospan	No arthritis
Baimukhamedov, 2021 [13]	Kazakhstan	F	38	VABIOTECH (Sputnik-V)	1st	20 d	Pain and stiffness in the shoulder, and swelling and pain in both knee joints	Moderate effusion	ESR 39 mm/h CRP 10 mg/L RF 170 IU/mL	RA	Methotrexate, methylprednisolone, and NSAIDs	N/A
Emran, 2022 [14]	USA	F	81	Moderna (mRNA-1273)	2nd	21 d	Diffuse joint pains, specifically right hand, and diffuse swelling	Mild right and left osteoarthritis of the distal interphalangeal joint and the first carpometacarpal joints	ESR 104 mm/hr CRP 4.8 mg/dL	Erosive arthritis and seronegative RA	Methotrexate	ESR declining and improvement in symptoms with residual swelling in the right wrist
Flowers, 2021 [15]	USA	F	68	N/A	N/A	7 d	Left shoulder pain, decreased range of motion, and fever	Large glenohumeral effusion with synovitis	Elevated inflammatory markers	SA	Subtotal bursectomy and antibiotics	N/A
Hyun, 2021 [9]	Korea	F	68	AstraZeneca-Oxford (AZD1222)	1st	3 d	Arthralgia in both hands and feet, fever, myalgia, and generalized edema	Increased uptake in both first MTP and left knee joints	ESR > 120 mm/h CRP 135 mg/L DD 3.76 µg/mL FEUs LDH 437 IU/L	Polyarthralgia and myalgia syndrome	NSAIDs and PDN	Symptoms relieved
			67			4 d	Arthralgia in both hands and ankles, fever, myalgia, and localized joint edema	Increased uptake in both shoulder, elbow, wrist, ankle, multiple fingers, first MTP, and left knee joints	ESR 32 mm/h CRP 13.1 mg/L DD 1.19 µg/mL FEUs LDH 1095 IU/L		NSAIDs	
			67			4 d	Arthralgia in both hands, feet, and shoulders, fever, myalgia, and joint edema	Increased uptake in both wrist, ankle, multiple fingers, first MTP, and right knee joints	ESR 76 mm/h CRP 41.3 mg/L DD 2.16 µg/mL FEUs LDH 490 IU/L		NSAIDs	
			25			3 d	Arthralgia in both hands and legs, fever, myalgia, and generalized edema	Not done	ESR 64 mm/h CRP 18.5 mg/L DD 3.18 µg/mL FEUs LDH 457 IU/L		NSAIDs	
			70			7 d	Arthralgia in the neck, back, both elbows, knees, hands, and feet, fever, chill, myalgia, and generalized edema	Increased uptake in the cervical spine, both wrist, hands, fingers, and left foot	ESR 60 mm/h CRP 293 mg/L DD 2.44 µg/mL FEUs		NSAIDs	
Lourenço, 2022 [16]	Portugal	F	75	AstraZeneca-Oxford (AZD1222)	1st	14 d	Limited active range of motion of both shoulders and hips and pain in the specific shoulder	Effusion on both shoulders, hips, and knees	ESR 120 mm/h CRP 80 mg/L	Polymyalgia rheumatica	Corticosteroids and PDN	Controlled pain, normal analysis, and ADL independence
Massel, 2021 [17]	USA	F	68	Pfizer	N/A	3 d	Dull left shoulder pain and decreased range of motion	Severe subacromial/subdeltoid bursitis	CRP 24.2 mg/L	SA	Intravenous vancomycin, ceftriaxone, IV ceftriaxone, and oral antibiotics	N/A
Padiyar, 2022 [18]	N/A	F	20	AstraZeneca-Oxford (AZD1222)	1st	10 d	High fever, generalized myalgia, ankle arthralgia, and evanescent maculopapular rash over the neck, abdomen, and thigh	Normal	ESR 51 mm/h CRP 38 mg/L	AOSD	Naproxen	Asymptomatic
		F	47			3 w	High fever and small and large joint inflammatory arthritis	Normal	ESR 86 mm/h CRP 169 mg/L		Methotrexate and tocilizumab	Remarkable improvement
		F	35			3 m	High fever, evanescent skin rash on the trunk/extremities, and arthralgias	Mild FDG avid bilateral cervical lymphadenopathy in Level I, II, III, and Level V nodes	ESR 48 mm/h CRP 227 mg/L		MP Pulse and tocilizumab	Improved
Park, 2021 [19]	Korea	F	36	Pfizer-BioNTech (BNT162b2)	1st	10 d	High fever, chilling sense, sore throat, and multiple joint pain including hands and ankles	Splenomegaly	ESR 87 mm/h CRP 18.95 mg/L	AOSD	Methylprednisolone and tocilizumab	Dramatic improvement, bilateral pleural effusion and cardiomegaly disappearing
Park, 2022 [20]	USA	F	73	Moderna (mRNA-1273)	1st	0 d	Swelling of the left index finger and dactylitis of the right second digit with tenderness, warmth, and prominent swelling causing limited flexion of the finger	Subcutaneous edema	ESR normal CRP high sensitivity	IA	Celecoxib	80% improvement overall
		M	64	Moderna (mRNA-1273)	2nd	1 d	Pain and swelling of left second digit	Grade III synovitis with grade II Doppler signal	N/A	IA	Celecoxib and methylprednisolone injection	Pain level decreasing
Roux, 2022 [21]	France	F	30s	Pfizer-BioNTech (BNT162b2)	1st	3 d	Acute pain in the right buttock	Inflammatory joint edges and bone erosion	N/A	Unilateral sacroiliitis	Ibuprofen	Sacroiliitis resolved
Shimagami, 2022 [22]	Japan	F	90 s	Pfizer-BioNTech (BNT162b2)	2nd	1 d	Pain in extremities and chest	Tenosynovitis of LHB	IFN-β markedly elevated ESR 73 mm/h CRP 167 mg/L	New-onset persistent polyarthritis	PDN	Dramatic improvement
		M	70 s	Pfizer-BioNTech (BNT162b2)	1st	1 d	Severe, persistent pain in both shoulders and the lateral side of the thighs	Tenosynovitis of the LHB on both sides, polyarthritis of the fingers and hands, and fluid accumulation in the greater trochanteric bursae	ESR 69 mm/h CRP 37 mg/L	New-onset persistent polyarthritis	PDN	Marked improvement
Singh, 2022 [23]	India	F	late 50 s	Bharat Biotech (BBV152, Covaxin)	1st	7 d	Acute onset facial puffiness, severe pain and swelling of the bilateral elbow, wrist, metacarpophalangeal, and proximal interphalangeal joints	Bony nodules near both her elbow joints	IgE > 1000 IU/mL (>30%) CRP 47 mg/L Eosinophilia persisted, rising up	Refractory hypereosinophilia associated with seropositive RA with rheumatoid nodules	Methotrexate, PDN, and indomethacin	Eosinophil counts normalizing
Sweeney, 2021 [24]	Australia	M	53	AstraZeneca-Oxford (AZD1222)	1st	8 w	Worsening headaches, arthralgia, pharyngitis, rash, and drenching sweats	Mild splenomegaly without lymphadenopathy	ESR 17 mm/h CRP 56 mg/L	AOSD	Oral PDN	Asymptomatic
Enginar, 2021 [25]	Turkey	F	74	Sinovac (CoronaVac)	1st	2 d	Pain and swelling in the right hand	N/A	ESR 84 mm/h CRP 20.2 mg/dL	ReA	PDN	ESR and CRP values decreasing
		M	76		2nd	7 d	Pain and swelling in the left hand	N/A	ESR 85 mm/h CRP 11.2 mg/dL	ReA	PDN	Arthritis not recurring
Watanabe, 2022 [26]	Japan	M	53	Pfizer-BioNTech (BNT162b2)	2nd	4 w	Swollen and painful left knee joint	Diffuse knee effusion	CRP 8.45 mg/dL RF 1200 U/mL	RA	Tocilizumab	Complete remission
Vanaskova, 2022 [27]	Czech Republic	M	53	Pfizer-BioNTech (BNT162b2)	1st	3 d	Persisting swelling and pain in knee joint	Effusion in the left knee joint	CRP 91.8 mg/L	Acute ReA	Dexamethasone administered	Full recovery with no consequences
Lebowitz, 2022 [28]	US	M	49	Pfizer-BioNTech (BNT162b2)	1st	7 d	Painful rash and swelling of the right foot	N/A	ESR ↑ CRP↑	Keratoderma blenorrhagicum in the setting of RA triggered	Intravenous methylprednisolone	Improvement
Leone, 2021 [29]	Italy	M	36	AstraZeneca-Oxford (AZD1222)	1st	1 d	High fever, evanescent and not pruritic rash, and chest pain	Bilateral pleural effusion, posterior wall pericardial effusion, subcentimetric lymphadenopathy, and mild splenomegaly	ESR 85 mm/h CRP 188 mg/L	AOSD	Anakinra	Complete normalization of symptoms and blood tests
Sharabi, 2021 [30]	Israel	M	43	Pfizer-BioNTech (BNT162b2)	2nd	10 d	High fever, arthralgia of both knees, and reddish macular rash	Large right knee effusion, bilateral pleural effusion, and diffuse bilateral infiltrates	CRP 9.32 mg/dL WBC 12.5 K/μL	AOSD	Solumedrol and oral PDN	Resolution of arthritis and myalgia
		F	56			7 d	High fever, swelling of metacarpo-phalangeal, proximal inter-phalangeal joints, and pink maculopapular rash	Pleural effusion	CRP 30.0 mg/dL WBC 40.0 K/μL		PDN	Immediate clinical improvement
Phukan, 2021 [31]	UK	F	70	Pfizer-BioNTech (BNT162b2)	1st	4 d	Sore left arm	N/A	CRP 88 mg/L ESR 114 mm/h	Rheumatic immune-mediated inflammatory disease	PDN	Improved
		F	44	AstraZeneca-Oxford (AZD1222)	1st	2 d	Painful and swollen right ankle		CRP 78 mg/L			
Klabklay, 2022 [32]	Thailand	F	45	AstraZeneca-Oxford (AZD1222)	1st	2 d	Left shoulder pain, limited range of motion, and fever	N/A	Staphylococcus aureus	SA	Antibiotic	Gradual improvement of clinical symptoms
Risal, 2022 [33]	Nepal	F	47	AstraZeneca-Oxford (AZD1222)	1st	7 d	High fever, dry cough, erythematous rash, left elbow pain and swelling	Hepatosplenomegaly	CRP 150 mg/L	AOSD	PDN	Improvement of joint pain
Yonezawa, 2022 [34]	Japan	M	54	Pfizer-BioNTech (BNT162b2)	2nd	1 d	Swelling and pain in the joints	N/A	CRP 7.64 WBC 13,240/μL	RA	Methylprednisolone and iguratimod	Good clinical response
Matsuda, 2022 [35]	Japan	F	59	Pfizer-BioNTech (BNT162b2)	1st	7 d	High fever, polyarthralgia, salmon-pink eruption, and tenderness of the left cervical lymph nodes	Multiple lymphadenopath and splenomegaly	WBC 13,320/Μl CRP 12.8 mg/dL	AOSD	PDN and intravenous tocilizumab	Symptoms improved
		F	77	Pfizer-BioNTech (BNT162b2)	2nd	42 d	High fever, polyarthritis, and salmon-pink rash	No hepatosplenomegaly or lymphadenopathy	WBC 10,090/μL ESR 93 mm/h CRP 20–25 mg/dL	AOSD	Methylprednisolone	No relapse
		M	35	Moderna (mRNA-1273)	1st	24 d	Spiking fever, salmon-pink rash, and polyarthritis	N/A	WBC 15,350/μL ESR 120 mm/h CRP 10.52 mg/dL	AOSD	PDN	No relapse
Albertino, 2022 [36]	Brasil	M	44	AstraZeneca-Oxford (AZD1222)	1st	2 d	Fever, odynophagia, myalgia, arthralgia, right cervical lymphadenopathy, pain near the right scapula and diarrhea, and salmon-pink maculopapular rash	Pleural effusion and a small amount of free fluid in the pelvis	WBC 21,400/mL CRP 362.2 mg/L	AOSD	PDN	Marked improvement
Chua, 2022 [37]	China Taiwan	F	30	Moderna (mRNA-1273)	2nd	16 d	Generalized skin rashes, symmetrical arthralgia, and persistent fever	N/A	WBC 16,950/μL Hs-CRP 27.695 mg/dL	AOSD	Intravenous methylprednisolone and naproxen	Complete remission
Nahra, 2022 [38]	USA	M	71	Pfizer-BioNTech (BNT162b2)	2nd	1 d	Diffuse joint pain	N/A	ESR 89 mm/h CRP 12.5 mg/L	IA	PDN	Symptoms subsided
		M	74		1st	10 d	Polyarthralgia and rash	N/A	ESR 6 mm/h CRP 15 mg/L RF 74 IU/mL (2 m)	RA	PDN and leflunomide	Improved
Winichakoon, 2022 [39]	Thailand	F	31	AstraZeneca-Oxford (AZD1222)	3rd	10 d	Fever, myalgia, arthralgia, pleuropericarditis, leukocytosis, and transaminitis	N/A	ESR 88 mm/h CRP 263 mg/dL	AOSD	Dexamethasone	Returning to normal
Koh 2022 [40]	Taiwan, China	F	17	Pfizer-BioNTech (BNT162b2)	1st	7 d	Fever, myalgia, headache, recurrent swelling, and a painful left and right knee	Joint effusions in both knees, right retrocalcaneal bursitis, bone marrow edema in the left proximal tibia, and sacroiliitis	ESR 85 mm/h CRP 57.9 mg/L	Peripheral SpA	Sulfasalazine, oral PDN, and etanercept	ESR and CRP returned to normal, and no relapse two months later

WBC = white blood cell, DD = D-dimer, FEU = fibrinogen-equivalent unit, ESR = erythrocyte sedimentation rate, CRP = C-reactive protein, LDH = lactate dehydrogenase, RF = rheumatoid factor, PDN = prednisolone, NSAID = nonsteroidal anti-inflammatory drug, SpA = spondyloarthritis, SA = septic arthritis, RA = rheumatoid arthritis, IA = inflammatory arthritis, AOSD = adult-onset Still’s disease, ReA = reactive arthritis, ↑ = elevated.

**Table 2:** Types of arthritis in bilateral/unilateral groups.

Characteristics	Unilateral (*n* = 15)	Bilateral (*n* = 30)
Age (yr)	56 ± 16	54 ± 20
Gender (M/F)	5/10	10/20
Vaccine Doses (1st/2nd/3rd/N/A)	9/4/0/2	22/7/1/0
Small/Large/Both Joints	2/12/1	2/4/24
RF (Positive/Negative/N/A)	1/4/10	6/19/5

M = male, F = female, RF = rheumatoid factor, N/A = not applicable.

**Table 3:** Cases with abnormal autoimmune markers.

Author (Year)	Gender	Age	Onset	RF	ANA	ACPA	IFN-β	CCP	HLA-B27	Final Diagnosis
Baimukhamedov, 2021 [13]	F	38	20 d	+++	−	+	N/A	N/A	N/A	RA
Emran, 2022 [14]	F	81	21 d		+	N/A	N/A	N/A	N/A	Erosive arthritis and seronegative RA
Shimagami, 2022 [22]	F	90s	1 d	+	−	N/A	+++	N/A	N/A	New-onset persistent polyarthritis
Singh, 2022 [23]	F	late 50s	7 d	+++	−	+++	N/A	N/A	N/A	Refractory hypereosinophilia associated with seropositive RA with rheumatoid nodules
Watanabe, 2022 [26]	M	53	4 w	+	N/A	+++	N/A	N/A	N/A	RA
Risal, 2022 [33]	F	47	7 d	−	+	−	N/A	N/A	N/A	AOSD
Yonezawa, 2022 [34]	M	54	1 d	+	−	−	−	N/A	N/A	RA
Matsuda, 2022 [35]	F	59	7 d	−	+	N/A	N/A	−	N/A	AOSD
	F	35	24	+	−	−		−	N/A	AOSD
	F	77	42 d	−	++	−	−	N/A	N/A	AOSD
Chua, 2022 [37]	F	30	16 d	−	++	−	−	N/A	N/A	AOSD
Nahra, 2022 [38]	M	71	1 d	−	++	N/A	N/A	−	N/A	IA
	M	74	10 d	+	N/A	N/A	N/A	N/A	N/A	RA
Koh, 2022 [40]	F	17	7 d	−	−	N/A	N/A	N/A	+	Peripheral SpA

+++ = strong positive, ++ = positive, + = weakly positive, − = negative, N/A = not applicable, M = male, F = female, HLA-B27 = human leukocyte antigen B27, ANA = antinuclear antibodies, ACPA = anti-citrullinated peptide antibodies, CCP = cyclic citrullinated peptide, RF = rheumatoid factor, IFN-β = Interferon-beta, SpA = spondyloarthritis, RA = rheumatoid arthritis, IA = inflammatory arthritis, AOSD = adult-onset Still’s disease.

**Table 4:** Quality assessment of the included studies.

Study	Selection				Comparability	Outcome			Score
	Representativeness of Exposed Sample	Selection of Nonexposed Sample Size	Ascertainment of Exposure	Demonstration That Outcome of Interest Was Not Present at Start of Study	Control for Important Factors/Additional Factors	Assessment of Outcome	Follow-Up	Adequacy of Follow Up	
An, 2021 [11]	0	1	1	1	2	1	1	1	8
Baimukhamedov, 2021 [12]	0	1	1	1	2	1	1	1	8
Baimukhamedov, 2021 [13]	0	1	1	1	2	1	1	1	8
Emran, 2022 [14]	0	1	1	1	2	1	1	1	8
Flowers, 2021 [15]	0	1	1	1	2	1	1	1	8
Hyun, 2021 [9]	1	1	1	1	2	1	0	0	7
Lourenço, 2022 [16]	0	1	1	1	2	1	1	1	8
Massel, 2021 [17]	0	1	1	1	2	1	0	0	6
Padiyar, 2022 [18]	1	1	1	1	2	1	1	1	9
Park, 2021 [19]	0	1	1	1	2	1	1	1	8
Park, 2022 [20]	0	1	1	1	2	1	1	1	8
Roux, 2022 [21]	0	1	1	1	2	1	1	1	8
Shimagami, 2022 [22]	0	1	1	1	2	1	0	0	6
Singh, 2022 [23]	0	1	1	1	2	1	1	1	8
Sweeney2021 [24]	0	1	1	1	2	1	1	1	8
Enginar, 2021 [25]	0	1	1	1	2	1	1	1	8
Watanabe, 2022 [26]	0	1	1	1	2	1	1	1	8
Vanaskova, 2022 [27]	0	1	1	1	2	1	1	1	8
Lebowitz, 2022 [28]	0	1	1	1	2	1	0	0	6
Leone, 2021 [29]	0	1	1	1	2	1	1	1	8
Sharabi, 2021 [30]	0	1	1	1	2	1	0	0	6
Phukan, 2021 [31]	0	1	1	1	2	1	0	0	6
Klabklay,2022 [32]	0	1	1	1	2	1	1	1	8
Risal, 2022 [33]	0	1	1	1	2	1	1	1	8
Yonezawa, 2022 [34]	0	1	1	1	2	1	0	0	6
Matsuda, 2022 [35]	1	1	1	1	2	1	1	1	9
Albertino, 2022 [36]	0	1	1	1	2	1	1	1	8
Chua, 2022 [37]	0	1	1	1	2	1	1	1	8
Nahra, 2022 [38]	1	1	1	1	2	1	1	1	9
Winichakoon, 2022 [39]	0	1	1	1	2	1	1	1	8
Koh, 2022 [40]	0	1	1	1	2	1	1	1	8

## Data Availability

Data will be made available on request.
